# Exploring mentorship as a strategy to build capacity for knowledge translation research and practice: a scoping systematic review

**DOI:** 10.1186/s13012-014-0122-z

**Published:** 2014-09-25

**Authors:** Anna R Gagliardi, Fiona Webster, Laure Perrier, Mary Bell, Sharon Straus

**Affiliations:** University Health Network, Toronto, Canada; University of Toronto, Toronto, Canada; St. Michael’s Hospital, Toronto, Canada; Sunnybrook Health Sciences Centre, Toronto, Canada

**Keywords:** Knowledge translation, Mentorship

## Abstract

**Background:**

Knowledge translation (KT) supports use of evidence in healthcare decision making but is not widely practiced. Mentoring is a promising means of developing KT capacity. The purpose of this scoping systematic review was to identify essential components of mentoring that could be adapted for KT mentorship.

**Methods:**

Key social sciences and management databases were searched from January 2002 to December 2011 inclusive. Empirical research in non-healthcare settings that examined mentorship design and impact for improving job-specific knowledge and skill were eligible. Members of the study team independently selected eligible studies, and extracted and summarized data.

**Results:**

Of 2,101 search results, 293 were retrieved and 13 studies were eligible for review. All but one reported improvements in knowledge, skill, or behavior. Mentoring program components included combining preliminary workshop-based training with individual mentoring provided either in person or remotely; training of mentors; and periodic mentoring for at least an hour over a minimum period of six months. Barriers included the need for infrastructure for recruitment, matching, and training; lack of clarity in mentoring goals; and limited satisfaction with mentors and their availability. Findings were analyzed against a conceptual framework of factors that influence mentoring design and impact to identify issues warranting further research.

**Conclusion:**

This study identified key mentoring components that could be adapted for KT mentorship. Overall, few studies were identified. Thus further research should explore whether and how mentoring should be tailored to baseline knowledge or skill and individual KT needs; evaluate newly developed or existing KT mentorship programs based on the factors identified here; and examine whether and how KT mentorship develops KT capacity. The conceptual framework could be used to develop or evaluate KT mentoring programs.

## Background

### Knowledge translation

Despite widespread focus on quality in the healthcare sector, performance reports highlight the need to improve the organization, delivery, and outcomes of healthcare services and programs [1]. Knowledge translation (KT) refers to an approach for improving healthcare and its outcomes by promoting and supporting the use of research in clinical, management, and policy-level decision-making [2]. KT includes knowledge transfer or dissemination, which refer to unidirectional sharing of research through mechanisms such as publications or presentations. KT also includes knowledge exchange, which refers to multidirectional interaction among researchers and decision makers for the conduct of research, program planning, evaluation, or quality improvement [3]. KT is a complex, multi-step, cyclical process that involves synthesizing evidence and creating knowledge products; interacting with target users to assess needs and identify barriers; using that information to tailor evidence syntheses or knowledge products and select implementation strategies; applying implementation strategies; and monitoring to evaluate impact and ensure that research use is sustained [2]. KT is a practice that can be applied by those interested in implementing newly generated evidence, or improving healthcare and its outcomes by promoting use of existing evidence. KT is also an evolving science that builds on theory and approaches from other disciplines to generate evidence on factors that influence research use, or on approaches, strategies and interventions that effectively support research use [4].

### KT capacity

KT capacity is needed to better equip those interested or engaged in KT practice or science. Decision makers, including clinicians, managers and policy makers, have professed that they are unfamiliar with, or confused about the concept and practice of KT, which may be why KT is not widely practiced [5,6]. In Canada, a survey of 240 health services researchers, and another survey of 265 directors of research organizations found that the majority shared research findings by disseminating them in print or electronic form [7,8]. Research in Australia and the United Kingdom identified the need to foster KT practice and research in nursing and primary care [9-12]. KT research was prioritized among practitioners and teachers of emergency medicine from 16 countries [13]. They acknowledged that few emergency medicine investigators have the skills to undertake KT research, and underscored the need to form linkages between emergency medicine clinician investigators and KT scientists to foster and support the conduct of KT research [14-16]. Interviews with individuals from 33 research funding agencies in Australia, Canada, France, the Netherlands, Scandinavia, the United Kingdom, and United States revealed a widespread need to increase our understanding and practice of KT [17]. We interviewed 18 researchers, clinicians, managers and policy makers who described many KT challenges including lack of awareness of what KT is and how to achieve it, little infrastructure for KT, and the absence of incentives to plan for and engage in KT [18].

Clearly, both researchers and decision makers require knowledge and skill to engage in KT practice or science. Recognizing this need, the United States National Institutes of Health and Veterans Health Administration developed a five-day training institute for postdoctoral fellows to learn about dissemination and implementation [19]. It was intended to function as a train-the-trainer program where participants share their learnings with others at their home institutions. The program included large group discussion and interactive small group sessions. Evaluation of the inaugural program by its 35 participants revealed that most had shared information with colleagues and submitted new grant applications. To improve the program participants recommended additional content on research design and research team leadership, and one-on-one mentorship to reinforce learning. Mentorship was a component of the Implementation Research Institute offered at Washington University in the United States [20] and the KT Canada Strategic Training Initiative [21].

### Mentorship

Mentorship is an interactive, facilitative process meant to promote learning and development that is based on educational and social learning theories [22]. Mentoring has been studied within the context of large corporations where it is used for training, professional development, and succession planning [23]. Seminal research by Kram found that mentoring consists of support for both professional (sponsorship, exposure and visibility, coaching, protection, challenging) and psychosocial (role modeling, acceptance and confirmation, counseling, friendship) development, and typically proceeds through four stages: initiation, cultivation, separation, and redefinition [24]. Significant benefits are associated with mentorship. Mentees receive more promotions, have higher salaries, experience less stress and conflict, are more satisfied with their jobs and careers, and are less likely to leave their organizations compared with non-mentees [25,26]. These positive outcomes are associated with both formal (matches made by a third party) and informal (self-initiated) mentorship, and are sustained longitudinally compared with those not mentored [26-28]. Mentors also derive benefit from mentoring including satisfaction from helping others, creation of free time for alternate pursuits, organizational recognition or reward, and improved job performance through exposure to new ideas [29,30].

### Mentorship in healthcare

In healthcare, mentoring has largely been studied in the context of academic medicine where physician trainees or junior physician investigators are mentored by those more experienced for career and personal development [31]. The benefits of academic medicine mentorship include greater grant capture, peer-reviewed publications, faster academic promotion, and higher career satisfaction [32]. A systematic review of published research on academic mentoring described the attributes and actions of an academic mentor, and several personal, relational, and structural barriers of mentoring [33]. Interviews with mentor and mentee physicians revealed that, while most believed academic mentorship to be important, they experienced difficulty in establishing productive mentoring relationships [34]. Strategies to enhance mentorship suggested by interview participants included the development of formal mentorship initiatives, and mentorship training for mentors and mentees. Mentoring has also been used for academic mentorship of student and novice nurses [35-39]. A large proportion of nurses reported having one or more mentors who served a variety of formal and informal roles [40]. A systematic review of academic mentoring in nursing found that it was associated with greater job satisfaction and academic achievement [41]. Interviews with, and surveys of mentor and mentee nurses emphasized the need for greater clarity of objectives, and strategies to identify and train mentors [42-44]. Mentoring was also recognized within the nursing literature as an important mechanism for research training in both university and clinical settings though the details of mentoring program design were not described [45,46].

### KT mentorship

Research funders, educators, investigators, and decision makers worldwide have identified the need to improve the quality of healthcare by building capacity for KT. Training programs for KT practice and science are few. Mentorship is a strategy that enhances access to knowledge which could be used alone, or in conjunction with KT training programs to develop KT capacity. However, limited empirical research has examined its application in healthcare, particularly outside of academic mentorship for physicians or nurses. Mentorship has been widely used in disciplines other than healthcare. The purpose of this research was to review literature in management and social sciences and identify essential components of mentoring programs that could be adapted for KT mentorship. More specifically, this review described the quantity and quality of research employing a variety of designs on mentorship, and examined the effectiveness of mentorship when used to develop job-related knowledge and skills among professionals in non-healthcare settings.

## Methods

### Approach

We conducted a scoping review of published research in the social sciences and management disciplines [47]. While similar in rigor to a traditional systematic review, a scoping review addresses broader, more complex topics where different study designs may be relevant and selection criteria are developed *post hoc* based on increasing familiarity with the literature [47]. A scoping review examines the way strategies, in this case mentorship, are used and their effectiveness, but also describes the nature of the literature to identify whether sufficient evidence exists to support a full systematic review. A scoping review also identifies gaps in the literature that can only be addressed through ongoing research. Preferred Reporting Items for Systematic Reviews and Meta-Analyses (PRISMA) criteria guided reporting of the methods and findings [48]. A protocol for this review was not registered.

### Conceptual framework

**Figure 1:**
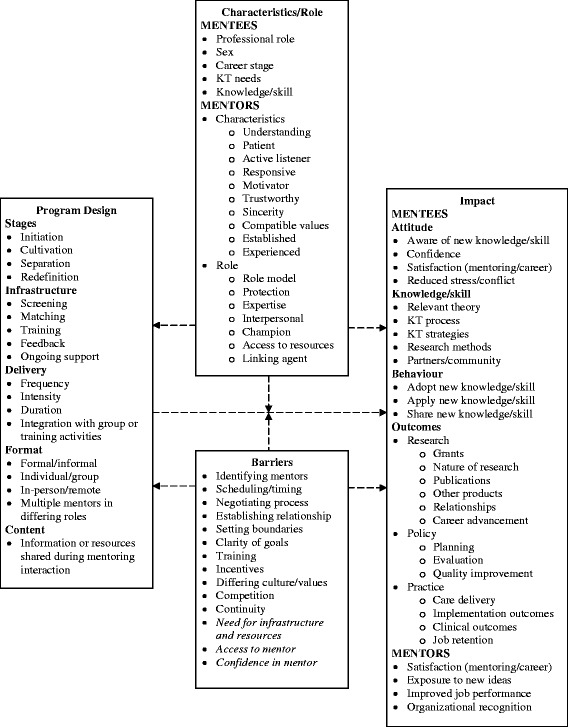
**Conceptual framework of factors influencing KT mentorship design and impact.** Italicized text represents elements not originally included in the framework that emerged from eligible studies

There is no single theory or model that describes mentorship processes, outcomes, and influencing factors. However, a useful framework for this study was developed by Karcher, who reviewed educational and psychology literature to outline elements of mentoring program design that might influence outcomes (goals, format, delivery, content, barriers) [22]. These elements, plus barriers of mentoring identified in our background review of research (identifying/securing mentors, scheduling, clarity of goals, negotiating process, preferences, training, stage-specific evaluation, incentives), and additional mentoring features identified by others were integrated with the Karcher framework [23-30]. The components of the conceptual framework (Figure 1) were used to interpret themes that emerged from the data. Unique themes were added to the conceptual framework.

### Data collection

Business Source Premier (BSP) and Social Sciences Citation Index (SSCI) were searched from January 2002 to December 2011 inclusive for empirical studies focusing on mentorship features and outcomes (Table 1). These represent key databases featuring the largest volume of published research in those disciplines. This date range reflected the most recent ten years of published research at the time of this review. Search strategies were purposefully broad to avoid eliminating potentially relevant items. They were developed by an information scientist with input from the study team. Search terms included mentor or coach as truncated terms to capture words stemming from these. The references of all eligible studies were also screened for relevancy. Mentorship was defined as any interactive process involving one or more mentors and mentees to promote professional development and/or improved professional performance related to specific knowledge or skills. Preliminary selection criteria included quantitative (surveys, observational studies, randomized trials), qualitative (interviews, focus groups) and mixed methods studies published in English language that focused on evaluating mentorship programs for the outcomes of knowledge and skills and provided sufficient detail to extract study design and findings. As search results were reviewed, selection criteria were expanded to specify studies that were not eligible. These included studies that focused on sport coaching or coach training, the development of psychosocial skills among mentees, or mentor characteristics, or were published in management or social sciences journals but studied mentorship in healthcare settings. Studies in the form of abstracts, letters, commentaries, or editorials were not eligible. Systematic reviews, though eligible in general, were excluded because they were conceptual or theoretical, focused on psychosocial benefits, characteristics of mentors or mentees, or did not evaluate mentorship program design. Titles and abstracts of search results were reviewed independently by two investigators and a research assistant. Titles and abstracts were re-screened by the principal investigator and a research assistant with refined eligibility criteria. Rather than calibrating agreement on initial selections, all items selected by at least one reviewer were retrieved for further assessment since judgment about eligibility must often be reserved until the full text can be reviewed. If more than one publication described a single study and each presented the same data, the most recent was included.

**Table 1:** **Search strategies**

**Source**	Search terms used*
Business Source Premiere	(mentor* OR coach*) NOT ((youth OR adolescen* OR teen* OR sport* OR athlet* OR football OR “high school*” OR basketball* OR golf*) OR Source (IEEE Transactions)). The following limits, available when searches were executed, were applied: Scholarly (Peer Reviewed) Journals; Publication Type: Academic Journal; Document Type: Article; Search modes - Boolean/Phrase
	Note: IEEE Transactions includes several publications issued by the Institute of Electrical and Electronics Engineers on electrical engineering, computing, biotechnology, telecommunications, power and energy which generated a high number of ineligible articles and were therefore excluded
Social Sciences Citation Index	(mentor* OR coach*) NOT (youth OR adolescent* OR teen* OR sport* OR athlet* OR football* OR “high school*” OR basketball* OR golf*) AND Language = (English) AND Document Types = (Article)

*Search strategies may not be replicable based on differing vendor systems and changes in search interfaces over time.

### Data extraction, analysis, and synthesis

A data extraction form was developed based on the conceptual framework to describe mentoring program goals, impact and design. The form was pilot tested independently by ARG and a research assistant for ten randomly selected articles. Extracted data were then compared to determine whether and how the form should be revised. Data were independently extracted by two research assistants from all articles using the updated form. Study quality was assessed using criteria relevant to study design, but was not used to exclude studies. This included the Cochrane Risk of Bias tool for randomized controlled trials, and a modified version of the Downs and Black instrument for observational studies [49,50]. Search results were depicted with a PRISMA flow diagram [48]. Extracted data were tabulated and the quantity, design, quality, and setting of studies were summarized. Studies included a wide range of designs, measures, and outcomes so pooling of data was not possible. Tabulated data were examined for trends or possible links between mentoring program design and outcomes to identify models that may be suitable for KT mentorship. Gaps in knowledge were also identified to issue recommendations for ongoing research.

## Results

### Study characteristics

**Figure 2:**
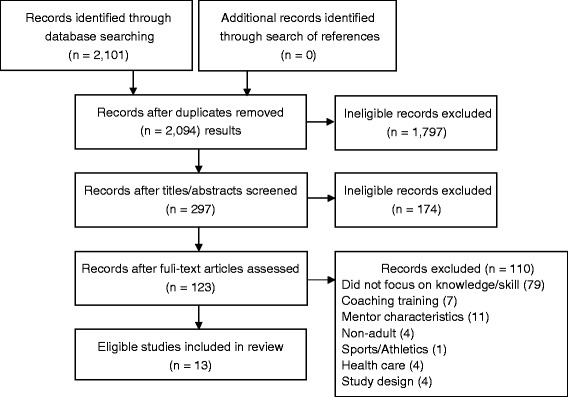
**PRISMA diagram of search results.**

The initial search resulted in 2,101 articles, of which 297 were retrieved as potentially relevant and further assessed. Of these, 13 studies (nine management, four social sciences) were eligible for review (Figure 2). Data are summarized in Table 2 [51-63]. At least one eligible article was published in eight of the ten years searched (other than 2006 and 2007) with a high of five published in 2009. More than half of the studies (seven) were conducted in the United States. Others were conducted in the United Kingdom (four), China (one), and Puerto Rico (one). Studies from management literature took place in manufacturing, tourism, educational institutions, financial services, service related industries, and non-profit settings. Studies from social sciences literature were predominantly based in educational environments including elementary and middle schools, and professional education programs.

**Table 2:** **Summary of mentorship studies identified in business and social science literature**

**Study**	**Research design (Quality)**	**Mentoring goal (improved or achieved)**	**Mentoring program design**							
			**Formal program**	**Individual or group**	**Senior mentor**	**Entry criteria**	**Mentor training**	**Dyad matching**	**Activities**	**Duration/Timing**
Elinger [51]	Survey	Self-reported overall job performance (yes)	No	—	Yes	No	No	No	—	—
2011/USA	(10/11)									
Wheeler [59]	Mixed	Company reported sales performance (yes)	No	—	Yes	No	No	No	—	—
2011/UK	(9/11)									
Kwan [52]	Survey	Self-reported overall job performance (yes)	Yes	Individual	Yes	No	No	No	—	—
2010/China	(11/11)									
Powell [60]	RCT (low risk of bias)	Teaching skills (yes, no difference between remote and on-site)	Yes	Both	Yes	No	Yes	No	2 day workshop, site visits	7 visits over 15 weeks, 90 min observation, 30 min discussion
2010/USA										
Agarwal [53]	Survey	Self-reported overall job performance (yes)	Yes	Both	Yes	Yes	Yes	No	—	—
2009/USA	(10/11)									
Bernal [63]	Mixed	Research skills (yes)	Yes	Individual	Yes	—	—	—	Consultation as needed	2 years
2009/Puerto Rico	(7/11)									
Fischer [54]	Mixed	Self-reported overall job performance (yes)	Yes	Individual	Yes	No	Yes	No	Mentor-mentee discretion	4 hours/month for 6 months
2009/USA	(10/11)									
Levenson [55]	Mixed	Self-reported overall job performance (no)	Yes	—	Yes	No	No	Yes	—	—
2009/USA	(8/11)									
Yost [61]	Mixed	Teaching skills (yes)	Yes	Individual	Yes	Yes	Yes	No	Workshop, site visits	3 visits/year, 45 min observation then discussion
2009/UK	(10/11)									
Byrne [56]	Survey	Self-reported overall job performance (yes)	Yes	—	Yes	No	No	No	—	—
2008/USA	(11/11)									
Stead [57]	Mixed	Self-reported overall job performance (yes)	Yes	Both	Yes	No	Yes	Yes	Workshops, meetings	Every 4 to 6 weeks over 9 months
2005/UK	(8/11)									
Browne-Ferrigno	Mixed	Teaching skills (yes)	Yes	Individual	No (peer)	—	—	—	Meetings	4 hours once per week over 8 months
[62] 2004/USA	(7/11)									
Wales [58]	Mixed	Self-reported overall job performance (yes)	Yes	Individual	Yes	No	Yes	Yes	Meetings	1 hour every 2 weeks over 1 year
2002/UK	(8/11)									

Quality assessment results appear in Table 2. The single randomized controlled trial had a low risk of bias. The 12 observational studies (eight mixed method program evaluations, four surveys) scored between 7 to 11 (median 9) on a scale of 11 with a higher score indicating higher study quality.

### Mentoring goals and impact

Studies examined impact of mentorship on self-reported overall job performance [51-58], and objectively assessed business performance [59], teaching skills [60-62], and research skills [63]. All studies reported achieving desired goals, or improving performance or skills except for two in which mentoring focused on job performance [55] and teaching skills [62].

### Mentoring design

Most studies examined formal, organized mentoring programs in which mentees were matched with mentors and/or specific mentoring activities were offered. Two studies examined informal mentoring programs in which the mentor-mentee relationship occurred spontaneously [51,59]. In six studies mentoring involved a single mentor and mentee [52,54,58,61-63], and in three studies mentoring combined individual and group activities [53,57,60], for example, participation in a two-day workshop followed by expert coaching [60]. Mentoring was hierarchical in most studies, meaning that the mentor was senior to the mentee or an expert. One study involved peer mentoring [62]. Two mentoring programs described eligibility criteria for mentees, for example, pre-requisite orientation prior to participation [53,61]. In six studies mentors were trained [53,54,57,58,60,61]. Mentoring programs matched mentors and mentees in three studies [55,57,58]. Of the seven studies that described mentoring program activities, three involved a training workshop followed by mentoring meetings or site visits [57,60,61] and four involved mentoring only [54,58,62,63]. In one of the mentoring-only studies, the frequency of meetings was left to the discretion of the mentor and mentee [63]. The duration of mentoring ranged from four months to two years. The frequency of mentoring meetings or site visits during specified periods was variable.

### Mentoring barriers

Two studies assessed challenges associated with mentoring [51,57]. Mentoring programs were resource intensive, requiring organizational support for sustainability and infrastructure for recruitment and matching. Feeling neglected by their mentor and mentor trustworthiness were also a concern for mentees. Others found that the goals were not clearly laid out by the mentorship program.

### Summary of eligible study findings

Data were examined to identify whether mentoring design features may be associated with impact. This also served to identify gaps in research. Of 13 studies, 12 reported achieving or improving desired knowledge, skills, or performance. Mentoring program design varied across these studies, as did the type of activities offered, and the duration and timing of mentoring in studies that reported these details. Therefore, potential associations were not identified. In the only study that did not achieve desired outcomes [55], the mentoring program design did not differ from that of studies in which mentoring had a positive impact. Another way to interpret these findings is that successful formal mentoring programs that are meant to enhance knowledge, skills, or performance may include individual and/or group mentoring offered by a senior or expert mentor. Mentor training may be required though entry criteria and dyad matching may not be necessary. Mentors and mentees should meet periodically over a given period of time from six months to one year, though optimal frequency is not clear. A preliminary workshop prior to mentoring can be used to transmit knowledge that is then reinforced by mentoring. However, it is notable that even informal mentoring with little infrastructure or processes achieved beneficial outcomes [51,59].

Data about design features and study findings were compared with components of the conceptual framework (Figure 1) to further identify gaps in research. Stages of mentoring were not addressed in eligible studies. Screening, matching, and training were described in some studies, though mentor training was most frequently addressed. Feedback and ongoing support were not addressed in eligible studies, however, preliminary training workshops were sometimes used to introduce concepts that were later reinforced by mentoring. Frequency, intensity, and duration were variable across studies in which mentoring had a positive impact. Most eligible studies involved formal mentoring, though two studies of informal mentoring also achieved positive results. Individual, and combined individual and group mentoring both resulted in beneficial outcomes. One trial found that mentoring delivered in person or remotely achieved similarly positive results. No studies involved or examined the roles of multiple mentors, ideal mentor characteristics, or outcomes experienced by mentors. This study focused on transfer of expertise so this was the only mentor role examined, and impact was limited to measures reflecting knowledge, skill and behaviour, most often by self-report. Barriers already included in the conceptual framework that were confirmed by eligible studies included clarity of goals. Additional barriers identified (italicized in Figure 1) included the need for resources and infrastructure, and issues related to the mentor-mentee relationship including feeling neglected by the mentor, and lack of confidence or trust in mentor expertise.

### Gaps in research

Recommendations for ongoing research were identified in two ways. First, data were examined to identify details that were often not reported by eligible studies. Two studies did not report information about matching of mentors and mentees, mentor training, or requirements of participation [62,63]. Several studies provided no details about mentoring activities, and duration and timing of mentoring [51-53,55,56,59]. Ongoing research that evaluates mentorship should more consistently report such details, and studies that develop, implement, and evaluate mentoring as an intervention should consider these design features when developing the mentoring program. Components of the conceptual framework not examined in eligible studies but that may be relevant to KT mentorship and warrant ongoing research includes stages of mentoring, mentor characteristics, roles and outcomes, and feedback and ongoing support. A concept revealed in eligible studies not already part of the conceptual framework that also warrants further research is how to blend group or training activities with mentoring. Neither the conceptual framework nor the eligible studies described the content of mentoring interactions. These unique concepts were added to the conceptual framework (Figure 1).

## Discussion

The purpose of this research was to identify essential components of mentoring used in management and social science applications that could form the basis of KT mentorship programs. Notably, all but one of the 13 eligible studies achieved desired outcomes or improvements in knowledge, skills, or associated behavior. These mentoring programs combined preliminary workshop-based training with individual mentoring; mentors received training and were either senior employees or external experts; and mentoring was offered for at least an hour periodically over a minimum of six months. One trial with positive results found that remote and in-person mentoring had similar impact. Some, but not all programs offered screening based on criteria or prerequisites, and mentor-mentee matching.

Interpretation and application of these findings are limited by several factors. We may not have identified all relevant studies despite employing a comprehensive search strategy and robust review methods. To enhance relevance of the findings for developing KT capacity we restricted the review to studies that focused on mentoring for job-specific knowledge or skills. Many of the search results focused on job satisfaction, organizational commitment and measures of career success such as promotion and salary. Such studies were not eligible so few studies were reviewed overall. While the quality of most studies was relatively high, the designs of these studies are more prone to bias in establishing a causal relationship between mentoring and the outcome of interest. Mentoring programs varied considerably in goals, design (frequency, intensity, duration) and measures of impact so pooling of data was not possible, nor would it be an option for future research, further limiting the inferences that can be drawn from this review. However, a strength of this study was use of a conceptual framework by which to analyze data, and identify gaps in knowledge and recommendations for future research, which is a key purpose for a scoping review.

KT training programs mentioned earlier [20,21] already include mentoring but published reports lacked details about this component, partially spurring this scoping review. Unfortunately, eligible studies lacked details about mentorship design and content. Ongoing research that evaluates mentorship should more consistently report such details, and studies that develop, implement, and evaluate mentoring as an intervention should consider these design features when developing the mentoring program. This has been advocated by others [64]. As a behavioural intervention, those interested in evaluating mentorship could draw upon established reporting guidelines combined with elements of the conceptual framework described here to more comprehensively address and then report design and content details [65,66].

Though only two of the eligible studies assessed barriers, several were identified, including the need for considerable infrastructure and resources to support activities such as recruitment, matching, and preliminary training events; lack of clarity in mentoring goals; and limited confidence in, and satisfaction with mentors and their availability. These barriers have also been identified by others, though in reference to academic mentoring [34,67]. Further research is needed to investigate optimal means by which to prevent or mitigate these issues. Such research might examine optimal training for KT mentors, and how to engage in remote mentoring as this may enhance access to mentors.

Several issues included in the conceptual framework were not addressed by eligible studies. This included stages of mentoring; and feedback and ongoing support. Ongoing studies that evaluate mentorship for developing KT capacity should explore whether and how complementary activities or mentoring content should be tailored according to baseline knowledge or skill, individual KT needs, and maturity of the mentoring relationship that reflects stages of mentoring. Such studies could also examine whether periodic interaction or other type of support may be needed following the formal period of mentorship to reinforce learning. No eligible studies examined mentor characteristics or outcomes. Given that the mentor characteristics may strongly influence the formation, function, and success of mentoring [34], further research should investigate the ideal characteristics of KT mentors. Eligible studies did not address the need for multiple mentors with differing roles. It has been recognized that those in influential roles may promote development or improvement in a variety of ways that include directing, teaching, championing or facilitating [68]. Roles may further differ based on the nature of the relationship. For example, coaching involves work-situated, specific and structured training over a finite period of time and is often delivered by a supervisor [69]. In contrast, mentoring is meant to address one or more personal, social, and professional needs through exchange of knowledge and sharing of experiences that may evolve over an extended period of time and can assume a variety of formats. The mentoring model may require one or more mentors with differing experience or over time to address multiple needs. Therefore further research could explore whether coaching, mentoring or both can best contribute to the development of KT capacity.

## Conclusion

Key components of mentoring programs that may form the basis of KT mentorship include combining preliminary workshop-based training with individual mentoring provided either in person or remotely; training of mentors; and periodic mentoring for at least an hour over a minimum period of six months. Further research is needed to address identified gaps includes developing and implementing, or evaluating existing KT mentorship programs based on the factors identified here, and examining whether and how KT mentorship develops KT capacity.
